# Women's Status and Violence against Young Married Women in Rural Nepal

**DOI:** 10.1186/1472-6874-11-19

**Published:** 2011-05-25

**Authors:** Prabhat Lamichhane, Mahesh Puri, Jyotsna Tamang, Bishnu Dulal

**Affiliations:** 1 1Center for Research on Environment Health and Population Activities, Kusunti, P.O. Box 9626, Kathmandu, Nepal

## Abstract

**Background:**

Studies conducted around the world consistently show the existence of violence against women. Despite the increasing number of studies being conducted on violence against young married women elsewhere, this subject has received little attention from researchers and policy makers in Nepal. This paper assesses the prevalence of violence among young married women in rural Nepal. Specifically, it examines [factors related to] women's status in order to better understand the risk of violence.

**Methods:**

A cross-sectional study was conducted in 2009 among 1,296 young married women aged 15-24 years in four major ethnic groups. Bivariate analysis and multivariate logistic regression were used to examine the association between selected risk factors and violence.

**Results:**

More than half the women (51.9%) reported having experienced some form of violence in their lifetime. One-fourth (25.3%) reported physical violence and nearly half (46.2%) reported sexual violence. Likewise, one-third (35.8%) of women reported experiencing some form of violence in the past 12 months. No or little inter-spousal communication and low autonomy of women significantly increases the odds of experiencing violence among married women.

**Conclusions:**

The violence against women is quite common among young married women in rural Nepal. Although the Domestic Violence and Punishment Act 2066 has been enacted, equal attention needs to be given to increasing women's autonomy and activities that encourage inter-spousal communication. Furthermore, more research is required in Nepal that examines dynamics of violence perpetrated by husbands.

## Background

Violence against women (VAW) has been recognized globally as a public health problem which violates human rights and incurs substantial social, economic and health costs. Though violence occurs in different forms and settings including workplace, school and community, violence at home 'domestic violence' is considered the most pervasive form [1]. Studies conducted across different countries have documented the widespread prevalence of domestic violence that includes range of physical, sexual and psychological acts perpetrated by intimate partners. Thirty five studies from different countries have revealed that one quarter to half of woman report having been physically abused by their present or former partner [2]. Likewise, a WHO multi-country study revealed 13% to 61% of women had experienced physical violence in their lifetime. Furthermore, in the majority of countries 10% to 50% of women reported being sexually abused [3]. A study conducted in Bangladesh reported that 67% of women reported experiencing domestic violence in their lifetimes and slightly more than one third of women reported experiencing violence in the past year [4].

Though the volume of evidence has been increasing globally, very few studies have been carried out in Nepal to assess the prevalence of violence in different settings and populations. A study conducted among female adolescents, youths and adults in eight districts of Nepal reported that more than one-third of married youths (20-24 years) experienced violence at home. A higher proportion of married youth reported experiencing violence compared to unmarried youth [5]. A qualitative study conducted among two major ethnic groups in Nepal revealed that sexual violence within marriage was common and the nature of the violence varied from verbal abuse to intimidation, beating to coercive sex [6].

Association between factors such as women's characteristics, partner's characteristics, and relationship status, and violence have been examined in past studies [3,4,7]. However, the limited number of studies conducted in developing countries to examine women's status and experience of violence show mixed results. For example, a study conducted in Bangladesh found that the association between women's status and domestic violence against women is highly context specific [8]. In the more culturally conservative area, higher women's individual autonomy and short-term membership in savings and credit groups were both associated with significantly elevated risks of violence. In contrast, in the less culturally conservative area, individual women's status indicators were unrelated to the risk of violence. Another study conducted in urban South India, found women's participation in social groups and vocational trainings to be positively correlated with the risk of violence [9]. Similarly, a study in Cambodia revealed that the frequency of spousal discussion and husband's control was positively correlated with violence [10].

However, to our knowledge no previous study has explored the potential association between women's status and VAW, particularly among married young women in rural Nepal. Nepalese culture, social and religious patterns, which enforce the lower status of women in family and society, may possibly act as a catalyst for violence. Social practices of early marriage limit the education and development of women. In addition, 'arranged marriage', in which family members help select the partner is still widely practiced than 'love marriage' in which people choose their own partner.

Furthermore, women are expected to play a subordinate, submissive and more conservative gender role in marital relationships especially in rural areas. Moreover, low status and low decision-making power of women, lack of access to resources, and information and shame in exposing certain abuses can put women at further risk of experiencing violence [11]. This is reflected in the poor health and social indicators of women in the country. For instance, the latest Demographic and Health Survey (2006) reported that only slightly more than half women were literate as compared to four-fifths of men [12]. A study on gender, caste and ethnic exclusion conducted in Nepal stated that health outcomes are directly affected by the subordinate status of women in the family [13]. Likewise, according to a Nepal Human Development Report, despite a declining trend in gender inequality, women's participation in political, economic and professional spheres remained lower than that of men [14].

This paper examines the association between women's status (including women's education, women's occupation, inter-spousal communication, women's autonomy and attitude of women towards justification of being hit) and VAW among young married women using data from a recently conducted cross-sectional study among four major ethnic groups from rural Nepal. We hope to contribute to the limited body of population-based research on VAW in Nepal and provide evidence to policy makers, program designers and public health professionals to make informed policies and programs.

## Methods

Data for this paper come from a cross-sectional survey, carried out in four districts - Dolkha, Sindhupalchowk, Dang and Kapilvastu of Nepal by the Centre for Research on Environment Health and Population Activities (CREHPA) in 2009. These districts were selected to represent four major ethnic groups and to reflect geographic variation and different levels of socio-economic development and cultural diversity of the country. The two groups from the hilly region (Brahmin/Chhetri and Tamang) and the two from the Terai region are Tharu and Muslims.

In the study 1,296 married women aged 15 to 24 years were interviewed using a two-staged systematic random sampling technique. In the first stage, 48 clusters in the selected districts were chosen using population proportionate to size. In the second stage, 27 households per cluster were selected after preparing an updated list of the households with the help of community leaders. After selecting a house, a short screening questionnaire was administered to the heads of the households (who generally make the decisions in household matters) to obtain basic information on all family members including age, sex and marital status. Based on the screening questionnaire, eligible respondents (married woman aged 15-24 years) were identified and selected using systematic random sampling. A total of 5,080 households were visited to identify eligible participants. Although all eligible women (1811) were identified in the visited households, the desired sample of 1,296 was interviewed. In cases of non-availability of eligible respondents, interviews were terminated after completing a short screening questionnaire. In households with more than one eligible woman, one woman was selected randomly for interview. Interviews were conducted individually at a convenient location for the respondents, usually outside their homes, by trained female interviewers. Twelve well-trained Nepali research assistants collected the data.

Most of the questions in the individual questionnaire administered to women were based on a 2005 WHO multi-country study [3] on woman's health and domestic violence against women which had been modified to suit the local context. The questionnaires were drafted in English and translated into Nepali, the national language of Nepal. All the translations were thoroughly checked to ensure that meaning of the original English version was retained. The core protocol and research instruments were approved by the ethical committees of the Nepal Health Research Council and the WHO Research Ethics Committee. Participants involved in the study were fully informed about the nature of the study, research objectives, and confidentiality of the data. Each participant's verbal consent was obtained regarding their participation in the study after assuring confidentiality. Interviewers orally provided contact information for organizations that address violence and conflict within marriage to all women. If any respondent ever experienced violence and sought help (either required counselling or other services) then interviewers facilitated access to an appropriate service facility or referred her to the nearest health centre. None of the eligible women refused to participate in an interview.

In this study, the definition of violence given by the WHO was adopted [15]. It is defined as "the intentional use of physical force or power, threatened or actual, against oneself, another person, or against a group or community that either results in or has a high likelihood of resulting in injury, death, psychological harm, maldevelopment, or deprivation". Respondents were asked if they had experienced different violent acts in their lifetime as well as in the past 12 months. The acts included to measure physical violence were:

• Slapped you or thrown something at you that could hurt you;

• Pushed you or shoved you or pulled your hair;

• Hit you with his fist or with something else that could hurt you;

• Kicked you, dragged you or beaten you up;

• Choked or burned you on purpose; and

• Threatened to use or actually used a gun, knife or weapon against you.

The acts included to measure sexual violence were:

• Physically forced you to have sexual intercourse with him even when you did not want to;

• Forced you to have sexual intercourse when you were afraid to say 'no' to sex;

• Threatened that if you didn't have sex with him he would leave you or go to another woman; and

• Forced you to do something sexual that you found degrading or humiliating.

Two dichotomous dependent variables were created to assess whether or not a woman has experienced violence in her lifetime and in the past 12 months by combining physical and sexual violence. These measures were operationalized for this study as "Experience of violence in lifetime" and "Experience of violence in the past year".

Independent variables included personal characteristics of woman, their husband, sex composition of children, media exposure, inter-spousal communication, women's attitude on 'men are justified to beat their wives', women's autonomy and household wealth status. Women's education was categorized into illiterate, literate upto primary, secondary and higher secondary and above. Women's occupation was trichotomized into housewife/no income, agriculture/daily wages/poultry farming (low income occupation) and other employment (service/small business). Woman were asked on whether or not they communicate with their husbands about seven matters including money matters, things that have happened to him in the day, things that have happened to her during the day, things that worry her, her feelings/love, things that worry him and his feelings/love. The responses were trichotomized into 'None', 'Discussed 1-3 matters' and 'Discussed 4-7 matters'. Women's attitudes on the idea that 'men are justified to beat their wives' was measured using six items including wife doesn't do household work, wife disobeys, wife refuses sex, wife asks about girlfriends, husband suspects that wife is unfaithful. A woman who believed that none of the six items justified violence was labelled as 'not accepting', a woman who believed that one to three of the items justified violence was labelled 'partially accepting' and a woman who believed that four to six items justified violence was labelled 'highly accepting'.

We constructed a variable to approximate women's status by applying latent class analysis. We considered women's autonomy to be an underlying (or latent) variable that could not be observed directly; it could, however, be measured through the observation of various manifest variables. We used 14 manifest variables covering household decision-making, control of resources, household communications, and autonomy/mobility. We used posterior probabilities of class membership in order to assign each woman a modal class. Our analysis included three latent classes for women's autonomy. Women belonging to class 1 (highest autonomy) were most likely to be able to make their own decisions concerning allocation of household resources, contraception, medication, visits to their maternal family and group membership, as well as being most likely to be able to communicate freely with their husbands on a variety of topics. Women belonging to class 2 (medium autonomy) are generally not involved with household decision making, except in terms of contraception, and tend to have some restrictions on things like becoming a member of community groups, but they still do well in terms of communication and tend to be free to visit friends and family. Women in class 3 (lowest autonomy) are least likely to be involved with any kind of family decision making, even in terms of their own health; women in this group also feel less able to communicate with their husbands. Women were assigned to a class on the basis of posterior probabilities of class membership based on their responses to the 14 manifest variables. This method allows the woman to be separated into different groups on the basis of the underlying latent variables and allows us to see the extent to which women's autonomy affects the likelihood of experiencing violence. The household wealth index was generated from 13 indicators on household dwelling and assets. Principal component analysis was used to generate "Household Wealth Index".

It was hypothesized that the following woman's status related factors would be associated with the increased risk of violence: ethnicity, low education level of woman, unemployed status of woman, lack of communication ability, women's accepting attitude on 'men are justified to beat their wives' and woman's autonomy. These factors were selected on the basis of literature that has examined dimensions of women's status, the association of women's status and violence and country context [16-19]. In addition, the qualitative research conducted among Tharu and Brahmin ethnicities in rural Nepal reported that 'lack of education', women's inability to negotiate with their husbands', 'traditional norms and cultural values' and 'lack of cash income' were perceived as the reason for sexual violence [6].

The dependent variable "Experience of violence in the past 12 months" was used for bivariate and multivariate analysis. The crude association between response and outcomes variables was assessed by using chi-square tests. Due to the dichotomous nature of the dependant variable, binary logistic regression was employed. In order to assess the association between women's status and VAW, we have controlled the effect of a number of factors in the model. The variables that were controlled for include current age of woman; age at marriage; type of marriage; sex composition of children; media exposure; wealth status; husband's age, education, occupation and alcohol use. Statistical Package for Social Sciences (SPSS v 13) was used to perform the data analysis.

## Results

### Characteristics of the study population

The median age of women was 22 and more than three-fifths (63.2%) of women were 21- 24 years of age (Table 1). The median age at marriage was 17 years. Nearly three-fourths (70.1%) of the women had 'arranged marriage'. Twenty eight percent of the women were illiterate. Nearly half (46.4%) of women had at least one son. More than three-quarters (78.6%) of the women were housewives and had no cash income. Most women (86.5%) had discussed 4-7 matters with their husbands. More than half (57.3%) of the women had no or rare exposure to the mass media. Over three-quarters of the women (77.9%) had "partially accepting" attitudes on men are justified to beat their wives. Overall, one-fourth of the women were in the lower autonomy class while 37.7% were in the higher autonomy class.

**Table 1:** Descriptive Characteristics of Young Married Women aged 15-24 years and association with lifetime experiences of violence, Rural Nepal, 2009

	All women *(N = 1296)*		Women with lifetime experiences of violence *(N = 673; 51.9%)*		Women without lifetime experiences of violence *(N = 623; 48.1%)*	
	**%**	**n**	**%**	**n**	**%**	**n**

Current age of women						
15-17	6.1	79	6.1	41	6.1	38
18-20	30.7	398	31.2	210	30.2	188
21-24	63.2	819	62.7	422	63.7	397
Age at marriage***						
10-14	7.0	91	8.5	57	5.5	34
15-17	51.9	672	55.3	372	48.2	300
18-24	41.1	533	36.3	244	46.4	289
Type of marriage						
Arranged marriage	70.1	908	70.4	474	69.7	434
Love marriage	29.9	388	29.6	199	30.3	189
Caste/ethnicity						
Muslim	25.0	324	25.9	174	24.1	150
Tamang	25.0	324	22.9	154	27.3	170
Tharu	25.0	324	26.7	180	23.1	144
Brahmin/Chhetri	25.0	324	24.5	165	25.5	159
Women's Education*						
Illiterate	28.6	371	29.1	196	28.1	175
Literate up to primary	51.2	664	53.6	361	48.6	303
Secondary	16.4	212	14.6	98	18.3	114
Higher secondary & above	3.8	49	2.7	18	5.0	31
Sex composition of children						
Childless	27.2	352	26.4	178	27.9	174
Only daughter	26.5	343	24.7	166	28.4	177
At least one son	46.4	601	48.9	329	43.7	272
Women's occupation						
Do not earn/housewife	78.6	1019	77.9	524	79.5	495
Agriculture/daily wages/poultry farming	17.0	220	18.6	125	15.2	95
Other employment (service/small business)	4.4	57	3.6	24	5.3	33
Inter spousal communication***						
None	4.3	56	7.1	48	1.3	8
Discussed 1-3 matters	9.2	119	10.8	73	7.4	46
Discussed 4-7 matters	86.5	1121	82.0	552	91.3	569
Media exposure						
No/rare	57.3	742	58.8	396	55.5	346
Frequent	42.7	554	41.2	277	44.5	277
Women's attitude on 'Men are justified to beat their wives'*						
Not accepting	12.4	161	10.5	71	14.4	90
Partially accepting	77.9	1009	78.0	525	77.7	484
Highly accepting	9.7	126	11.4	77	7.9	49
Women autonomy***						
Low	25.0	324	28.7	193	21.0	131
Medium	37.3	484	38.9	262	35.6	222
High	37.7	488	32.4	218	43.3	270
Household wealth status						
Poorest	19.3	250	18.9	127	19.7	123
Poor	20.4	265	21.2	143	19.6	122
Middle	20.0	259	19.9	134	20.1	125
Rich	20.1	261	21.0	141	19.3	120
Richest	20.1	261	19.0	128	21.3	133

Chi-square test significant at * p ≤ 0.05, *** p ≤ 0.001

Among the women's characteristic, age at marriage, women's education, inter-spousal communication, women's opinion on 'men are justified to beat their wives' and women's autonomy was significantly associated with women's lifetime experiences of violence. The proportion of illiterate women among women with lifetime experiences of violence and those without lifetime experience of violence was almost equal (29.1% vs 28.1%). The percentage of woman who did not discuss any matters with husband was higher among women who experienced violence in lifetime than those who did not. Similarly regarding women's autonomy, 25% were from low autonomy class; this percentage was higher among women who experienced violence in lifetime than those who did not experience violence (28.7% vs 21%).

The mean age difference between husband and wife was 3.35 years and almost half of their husbands were below 24 years of age (Table 2). More than one-tenth (13.9%) of the husbands were illiterate and one-tenth (10.7%) reported an education level higher than secondary level or above. Agriculture was the main occupation for one-fourth of the husbands. About 16 percent of women reported that their husbands consumed alcohol often. Husband's age, education and alcohol was found to be significantly associated with lifetime experience of violence.

**Table 2:** Husband's characteristics of young married women aged 15-24 years and association with lifetime experiences of violence, Rural Nepal, 2009

	**All women ***(N = 1296)*		Women with lifetime experiences of violence *(N = 673; 51.9%)*		Women without lifetime experiences of violence *(N = 623; 48.1%)*	
	**%**	**n**	**%**	**n**	**%**	**n**

Current age of husband**						
15-24	49.7	644	49.0	330	50.4	314
25-34	47.4	614	46.7	314	48.2	300
35+	2.9	38	4.3	29	1.4	9
Husband's education***						
Illiterate	13.9	180	15.8	106	11.9	74
Literate up to primary	53.6	695	57.1	384	49.9	311
Secondary	21.8	282	18.6	125	25.2	157
Higher secondary and above	10.7	139	8.6	58	13.0	81
Husband's occupation						
Unemployed	4.9	63	4.8	32	5.0	31
Agriculture	26.6	345	29.1	196	23.9	149
Daily wage labor	20.1	261	21.5	145	18.6	116
Services	21.1	274	19.9	134	22.5	140
Foreign employment	20.1	260	18.0	121	22.3	139
Business	7.2	93	6.7	45	7.7	48
Husband's alcohol use***						
Often	15.7	203	19.5	131	11.6	72
Sometimes	22.4	290	22.7	153	22.0	137
Never/rarely	62.0	803	57.8	389	66.5	414

Chi-square test significant at ** p ≤ 0.01, *** p ≤ 0.001

### Prevalence and nature of violence

Overall, more than half of young married women (51.9%) reported having ever experienced some type of violence from their husbands (Table 3). Nearly half (46.2%) reported sexual violence and one-fourth (25.3%) reported physical violence. Nearly one in five (19.6%) women reported ever experiencing both sexual and physical violence. In terms of sexual violence experienced in a woman's lifetime, physically forcing her to have sexual intercourse when she did not want to was the most commonly reported act (44.9%). About one-fourth (24.8%) of women reported ever experiencing forceful sexual intercourse when they were afraid to say 'no'. More than one in ten women reported that the husband had kicked, dragged or beaten them.

**Table 3:** Prevalence of violence against young married women according to type of violence, Rural Nepal, 2009

Type of violence	Lifetime % (N = 1296)	Past 12 months % (N = 1296)
Slapped you or thrown something at you that could hurt you	15.0	9.3
Pushed you or shoved you or pulled your hair	15.7	11.4
Hit you with his fist or with something else that could hurt you	5.9	3.7
Kicked you, dragged you or beaten you up	11.0	8.1
Choked or burnt you on purpose	0.9	0.6
Threatened to use or actually used a gun, knife or other weapon against you	0.9	0.6
*Physical violence: at least one type*	*25.3*	*17.4*
Physically force you to have sexual intercourse with him even when you did not want to	44.9	29.7
Force you for sexual intercourse when you were afraid to say 'no' for sex because of fear that he would do something	24.8	19.0
Threaten you that if you didn't have sex with him he would leave you or go to another woman	9.8	6.9
Force you to do something sexual that you found degrading or humiliating	13.5	12.1
*Sexual violence: at least one type*	*46.2*	*31.3*
**Experienced either physical or sexual**	**51.9**	**35.8**

More than one-third of women (35.8%) reported experiencing violence in the past 12 months. Sexual violence was reported by 31.3% of women and physical violence was reported by 17.4%. The pattern of the nature of violence was similar between lifetime experience and experience in the past 12 months. Similar to lifetime experience, the most common violent act was forceful sexual intercourse with more than one-fourth (29.7%) of women reporting to have experienced the act in the past 12 months. More than one in ten (11.4%) reported that the husband pushed or shoved them or pulled their hair in the past 12 months.

### Association between selected factors and violence in the past 12 months

The association of women's status related factors with experience of VAW in the past 12 months was examined (Table 4). Nearly one-fourth of Brahmin/Chhetri women experienced violence in the past 12 months whereas the proportion was 47.2% among Tharu. Likewise, about two in five illiterate woman experienced violence in the past 12 months. One third (34.2%) of women who had no earnings or were housewives experienced violence in the past 12 months, whereas the proportion was nearly one fifth (19.3%) among women in service or small businesses. The results showed that women who had no spousal communication were at increased risk of experiencing violence than women who discussed 4-7 matters (75% vs. 31.9%). Regarding autonomy and experience of violence in the past 12 months, 45.1% of woman in lower autonomy class experienced violence whereas the proportion was 28.3% among woman in higher autonomy class.

**Table 4:** Percentage of young married women Nepal reporting experiences of violence in the past 12 months by selected characteristics, Rural Nepal, 2009

Characteristics of women	Past year %	N
Caste/ethnicity***		
Brahmin/Chhetri	23.1	324
Tamang	27.5	324
Tharu	47.2	324
Muslim	45.4	324
Women's education***		
Illiterate	39.1	371
Literate up to primary	38.1	664
Secondary	26.9	212
Higher secondary and above	18.4	49
Women's occupation***		
Do not earn/housewife	34.2	1019
Agriculture/daily wages/poultry farming	47.7	220
Other employment (service/small business)	19.3	57
Inter spousal communication***		
None	75.0	56
Discussed 1-3 matters	53.8	119
Discussed 4-7 matters	31.9	1121
**Men are justified to beat their wives**		
Not accepting	33.5	161
Partially accepting	35.2	1009
Highly accepting	43.7	126
Women's autonomy***		
Low	45.1	488
Medium	37.2	484
High	28.3	324
**Total**	**35.8**	**1296**

Chi-square test significant at * p ≤ 0.05; ** p ≤ 0.01; ** *p ≤ 0.001

### Multivariate analysis

The association between women's status related factors and experience of violence was assessed using logistic models for experience of violence in the past 12 months. The results are presented in Table 5.

**Table 5:** Odds ratio (and 95% confidence intervals) from binary logistic regression of selected risk factors with experience of violence ^a^

Characteristics of women	Experience of violence in the past 12 months	
	**OR**	**95.0% C.I**

**Ethnicity**		
Muslim	1.91	1.04- 3.53
Tamang	0.80	0.48- 1.32
Tharu	1.61	0.93- 2.78
Brahmin/Chhetri (Ref)		
**Women's Education**		
Illiterate	0.75	0.29- 1.97
Literate up to primary	1.11	0.45- 2.76
Secondary	1.17	0.48- 2.82
Higher secondary and above (Ref)		
**Women's occupation**		
Do not earn/housewife	1.44	0.68-3.02
Agriculture/daily wages/poultry farming	2.17	0.97- 4.85
Other employment (service/small business) (Ref.)		
**Inter spousal communication**		
None	6.82	3.45- 13.48
Discussed 1-3 matters	2.33	1.52- 3.58
Discussed 4-7 matters (Ref.)		
**Men are justified to beat their wives**		
Not accepting	0.61	0.34- 1.07
Partially accepting	0.72	0.47- 1.11
Highly accepting (Ref.)		
**Women's autonomy**		
Low	1.65	1.08- 2.5
Medium	1.88	1.33- 2.64
High (Ref.)		

^a ^Controlled for current age of women, age at marriage, type of marriage, sex composition of children, media exposure, household wealth status, current age of husband, husband's education, husband's occupation and husband's alcohol use

Similar to the bivariate results, Muslim women were significantly more likely to experience violence as opposed to Brahmin/Chhetri women. Women's education was not significantly associated with the experience of violence. However, experience of violence was significantly associated with women in low income occupations. The risk of violence was more than two-fold among women who were in agriculture or daily wage occupation as compared to women who were in service or small businesses. The correlation between women's education and women's occupation was investigated by rank correlation coefficients which did not reveal significant correlation (Spearman's rho= 0.032).

The odd of experiencing violence was six times higher among women who had no communication than woman who report discussing 4-7 matters with their husbands. Similar to the bivariate results, the multivariate results did not show an association between women's attitude of woman on 'men are justified to beat their wives' and experience of violence. However, women's autonomy was associated with experience of violence as women with lower autonomy were more likely to experience violence as opposed to women with the higher autonomy.

## Discussion

The prevalence of violence found in this study was similar to what was found in other developing countries as more than half of young women in rural Nepal experienced some type of violence in their lifetime [3]. Based on type, more women experienced sexual violence compared to physical and emotional violence. This was consistent with findings from a multi-country study in Ethiopia, Bangladesh and Thailand [3].

Our study focused on examining the association between women's status-related factors and the experience of violence. Women's autonomy and inter-spousal communication were revealed to be significant predictors of violence, whereas education was not. With regard to ethnicity, Muslim women were at a significantly higher risk of experiencing violence whereas Tamang woman were found to be at lower risk compared to other ethnicities. This may be due to the underlying socio-religious practices among Muslim women who are more restricted to their conservative gender roles as compared to Tamang and Brahmin woman.

Results did not show an association between education and experience of violence in the multivariate analysis. This finding is difficult to interpret and needs further exploration. Educated women may be less likely to accept traditional gender roles and could be more vocal in their response to violence which may be taken as a transgression in a male-dominated Nepalese society. Studies conducted elsewhere have also presented conflicting results with some studies showing higher education to be protective while others show higher education as a risk factor [17,20,21]. The lack of association seen in our results may also be explained by findings from other studies [22,23] which have shown that difference in educational level between husband and wife is responsible for violence experience by woman. When the variable for women's educational level was replaced with a relative educational attainment in the logistic regression model, the results showed that women with higher educational levels than their husbands were at higher risk of experiencing violence as compared to women whose husband had higher education than them.

Likewise, though unemployed women were expected to be at higher risk, results showed woman in agriculture/daily wages/poultry farming occupations to be at higher risk of experiencing violence in the past 12 months. Involvement of women in economic activities may have been considered a factor challenging women's propriety and modesty and it may have acted as provocative rather than protective factor. Inter-spousal communication was a strong predictor of violence experience in the past 12 months. Results showed that women with no or little communication faced a significantly higher likelihood of violence. Married at an early age, women in Nepal are expected to be submissive, quiet, disciplined and loyal to the husband. In addition, women may be less empowered to protect themselves against violence as a result of young age at marriage. This may result in ineffective communication with husbands. Studies conducted in Egypt and other countries have also reported the inability of women to effectively communicate their problems and misunderstandings as a trigger for sexual violence [24,25]. However, contrary to our research findings, a study from Cambodia reported higher odds of violence with increased communication between partners [9]. As expected, both the bivariate and multivariate results found that women's autonomy was a significant predictor of violence. Higher autonomy was protective of the violence whereas lower autonomy increased the odds of violence experience. These results are consistent with those in Bangladesh and South Africa [10,22].

Although this study provides the most comprehensive information to date on the prevalence and association of women's status with violence in Nepal, potential limitations of the study need to be acknowledged. First, because of the cross-sectional nature of the study design, neither causal nor temporal ordering of the associations can be inferred. Second, although the research assistants had been adequately trained in interviewing and rapport building; violence experience may have been underreported because of the sensitivity of the subject.

## Conclusions

Marriage is thought to protect women from violence. However, our study results demonstrate that VAW is a significant public health problem in rural Nepal affecting more than half of 'young married women' thus demonstrating the magnitude of the problem. The main focus of our analysis was on women's status related variables and experience of violence which revealed that no or little inter-spousal communication and low autonomy of women increased the odds of violence experience among young married women. Although the Domestic Violence and Punishment Act 2066 [26] has been enacted, these results suggest that equal attention needs to be given to activities improving women's autonomy. Educational campaigns focused on redefining the roles of women in the family and society may help improve the status of women, however, the possible repercussions of the proposed intervention needs to be well considered. Interventions promoting inter-spousal communication may help prevent violence perpetrated by husbands. Overall, further research is needed in Nepal to understand dynamics of women's status, its association with different socio-religious practices and experience of violence. In addition, longitudinal studies examining the status of women since marriage can provider better insight into the dynamics of violence.

## Competing interests

The authors declare that they have no competing interests.

## Authors' contributions

PL analyzed the data, interpreted the findings and prepared the manuscript. MP designed the study, developed study instruments, supervised the data collection and was involved in preparing the manuscript. JT designed the study, developed study instruments and supervised the data collection.BD was involved in statistical analysis. All authors read and approved the final content of the manuscript.

## Pre-publication history

The pre-publication history for this paper can be accessed here:

http://www.biomedcentral.com/1472-6874/11/19/prepub
